# Quasi-static and dynamic experimental studies on the tensile strength and failure pattern of concrete and mortar discs

**DOI:** 10.1038/s41598-017-15700-2

**Published:** 2017-11-10

**Authors:** Xiaochao Jin, Cheng Hou, Xueling Fan, Chunsheng Lu, Huawei Yang, Xuefeng Shu, Zhihua Wang

**Affiliations:** 1 State Key Laboratory for Strength and Vibration of Mechanical Structures, School of Aerospace Engineering, Xi’an Jiaotong University, Xi’an, 710049 China; 2 Department of Mechanical Engineering, Curtin University, Perth, Western Australia 6845 Australia; 3 Shanxi Key Laboratory of Material Strength and Structural Impact, Taiyuan University of Technology, Taiyuan, 030024 China

**Keywords:** Mechanical engineering, Mechanical properties

## Abstract

As concrete and mortar materials widely used in structural engineering may suffer dynamic loadings, studies on their mechanical properties under different strain rates are of great importance. In this paper, based on splitting tests of Brazilian discs, the tensile strength and failure pattern of concrete and mortar were investigated under quasi-static and dynamic loadings with a strain rate of 1–200 s^−1^. It is shown that the quasi-static tensile strength of mortar is higher than that of concrete since coarse aggregates weaken the interface bonding strength of the latter. Numerical results confirmed that the plane stress hypothesis lead to a lower value tensile strength for the cylindrical specimens. With the increase of strain rates, dynamic tensile strengths of concrete and mortar significantly increase, and their failure patterns change form a single crack to multiple cracks and even fragment. Furthermore, a relationship between the dynamic increase factor and strain rate was established by using a linear fitting algorithm, which can be conveniently used to calculate the dynamic increase factor of concrete-like materials in engineering applications.

## Introduction

Concrete-like materials have been playing an invaluable role in structures and constructions. During their service lives, concrete structures might be subjected to blast and impact loads. Therefore, understanding the mechanical properties of concrete-like materials under dynamic loading is of great importance. Over the past several decades, studies on the static and dynamic mechanical properties of concrete-like materials have been conducted, and it is shown that, with the increase of strain rate, both dynamic compressive strength^1,2^ and tensile strength^3–5^ significantly increase. The dynamic increase factor (DIF), defined as a ratio of the dynamic compressive/tensile strength to its quasi-static counterpart, has been widely adopted to evaluate the strain rate effect of concrete-like materials^6–8^. Here, it is worth noting that the increase of compressive strength with strain-rate is mainly due to inertial effects. Compared with dynamic compressive strength, dynamic tensile strength is harder to measure and the DIF trend is more complicated. Thus, there is still lack of an accurate formula for the estimation of DIF of concrete-like materials under dynamic tensile loading. Especially, when the strain rate is in the range of 1–200 s^−1^, DIF is difficult to be accurately determined^2,3,5,6,9,10^ since it is rapidly increasing and appears obviously higher than that at a lower strain rate (see Supplementary Fig. S1). Unfortunately, this is what just happens for construction structures under impact loadings (see Supplementary Fig. S2), and there are few relevant studies reported on their dynamic strength. Therefore, a further investigation on DIF in this range of strain rates is quite necessary.

In the last few decades, a series of methods have been developed to test the static and dynamic tensile strengths of concrete materials. Among these popular testing methods (e.g., direct tension, splitting tension, spall and three- or four-point bending), splitting tensile tests are normally carried out with the split Hopkinson pressure bar (SHPB)^9,10^. Compared with other methods, specimens for Brazilian disc (BD) splitting tests are convenient to prepare, and experiments are easily performed with high accuracy of results. In addition, the strengths within a wide range of strain rates can be obtained by dynamic splitting tests with SPHB. Therefore, BD splitting tests have become the most common indirect tensile testing method for concrete-like materials^11–13^. In the BD tests, pressure is applied on a disc specimen along its radial direction, which generates tensile stress at the direction vertical to loading, causing the specimen failure. Taking inertial and strain rate effects into account, DIF obtained from dynamic splitting tests may overestimate the dynamic strength of concrete-like materials and cause potential security problems of structures subjected to earthquakes, blasting and hard impact^2,6^. Previous results show that, when the ratio of thickness to diameter for a BD specimen is equal to 0.5, the inertial effect can be minimized and DIF is mainly contributed by the real strain rate effect^14–17^.

During dynamic splitting tension, the start-split location and failure pattern of specimens are main factors that affect the reliability of tests. Generally speaking, experimental results are valid only when an initial crack occurs at the center of a specimen and propagates along the loading diameter direction^18,19^. However, the compressive stress concentration near the loading platen has a significant influence on the results of BD tests. Therefore, it is necessary to investigate the stress distribution and failure process of a specimen under impact loading. To solve this problem, different loading methods were developed to reduce the stress concentration and ensure the crack initiation point located at the center of BD specimens^20,21^. Three different failure modes (i.e., a single crack, multiple cracks, fragmentation) of BD specimens under splitting loading were also summarized^22^. Additionally, closed full-field solutions of stress- and displacement-fields in BD specimens with different loading conditions were obtained, based on the two-dimensional elastic theory with an assumption of concrete-like materials being linearly elastic and homogeneous^23,24^.

Concrete and mortar, as two typical concrete-like materials, are widely used in engineering structures. However, most of previous studies were mainly focused on the dynamic tensile strength of concrete. Since mortar is prepared without coarse aggregates, its mechanical properties, failure pattern and DIF are significantly different from concrete, especially for tensile strength. There is still lack of a reliable relationship between the DIF and strain rate for mortar materials that are usually used to glue bricks together or as surface binders between metope and ground^19,25^. Thus, it is necessary to conduct further investigation to highlight their different failure modes and dynamic tensile strengths under various strain rates.

In this paper, the tensile strength and failure pattern of concrete and mortar were investigated under quasi-static and dynamic loadings based on splitting BD tests. The paper is organized as follows. In the section Methods, the experimental methods are introduced, including preparation of specimens and principles of compressive, quasi-static and dynamic splitting tests. Then, the failure mode and tensile strength under quasi-static and dynamic splitting tests are discussed in Results and Discussion, with the relationship between the DIF and strain rate. Finally, a brief summary is given in Conclusions.

## Methods

### Material and specimens

**Figure 1:**
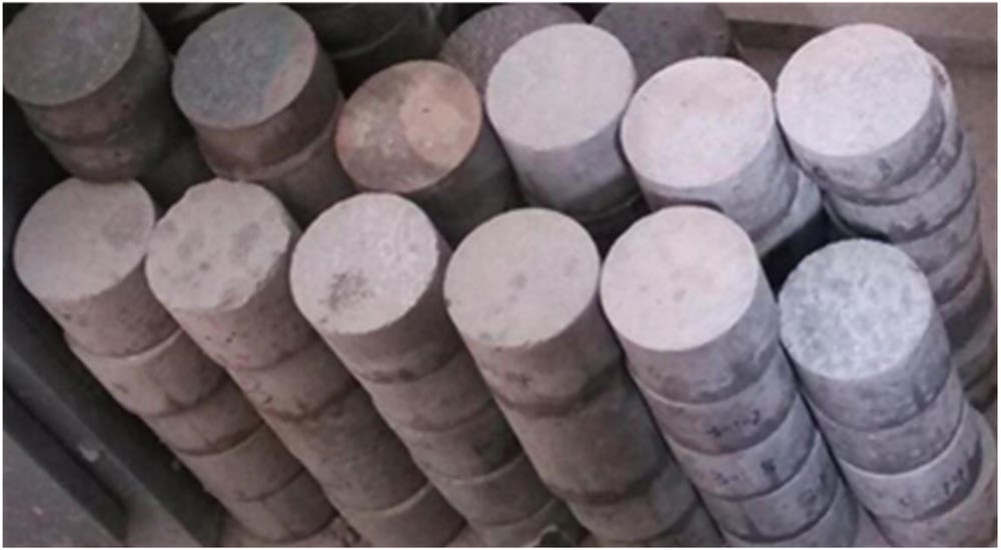
Photograph of the prepared BD specimens.

To study the influence of specimen size on dynamic splitting strength, both concrete and mortar BD specimens were cast in steel molds with two different sizes: diameter *D* = 70 mm and thickness *H* = 30 mm, *D* = 70 mm and *H* = 55 mm, respectively. Concrete was prepared in the weight proportion of 0.40:1.00:1.12:2.28 (water: cement: sand: coarse aggregate), while that of mortar was 0.58:1.00:3.03 (water: cement: sand). In preparation, crushed limestones were chosen as coarse aggregates, and fine aggregates were river sands. The sizes of coarse aggregates were smaller than 7.50 mm, with the apparent density, stacking density and porosity of 2.60 g·cm^−3^, 1.70 g·cm^−3^ and 37%, respectively. The sizes of sands were smaller than 2.36 mm, with the apparent density, stacking density and porosity of 2.55 g·cm^−3^, 1.45 g·cm^−3^ and 35%. Cement was chosen as the ordinary portland cement (OPC 32.5). In addition, to obtain quasi-static compressive strengths that are usually applied in the classification of concrete and mortar, three standard concrete and mortar specimens were cast in the shape of a block with dimensions of 150 × 150 × 150 mm^3^ and 70.7 × 70.7 × 70.7 mm^3^, respectively. These specimens were de-molded after 48 h, and then moist-cured in a standard curing room with temperature of 20 ± 2 °C and relative humidity of 95%. The prepared BD specimens are shown in Fig. 1.

### Compressive test

Before compression, the densities of concrete and mortar specimens were tested, and their average values are 2.40 and 2.02 g·cm^−3^, respectively. The compressive strengths were tested with the MTS testing machine, according to the standards for evaluation of concrete strengths (i.e., GB/T 50107-2010). For cube specimens, the compressive strength *f*_*c*_ can be obtained by

1 $${f}_{c}=\frac{{F}_{c}}{{A}_{c}}$$

where *F*_*c*_ and *A*_*c*_ are the failure load and cross-sectional area of a specimen. As a result, the average compressive strengths of three concrete and mortar specimens are 34.50 and 25.60 MPa; while their Young’s moduli are 30.32 and 27.45 GPa, respectively.

### Quasi-static splitting test

**Figure 2:**
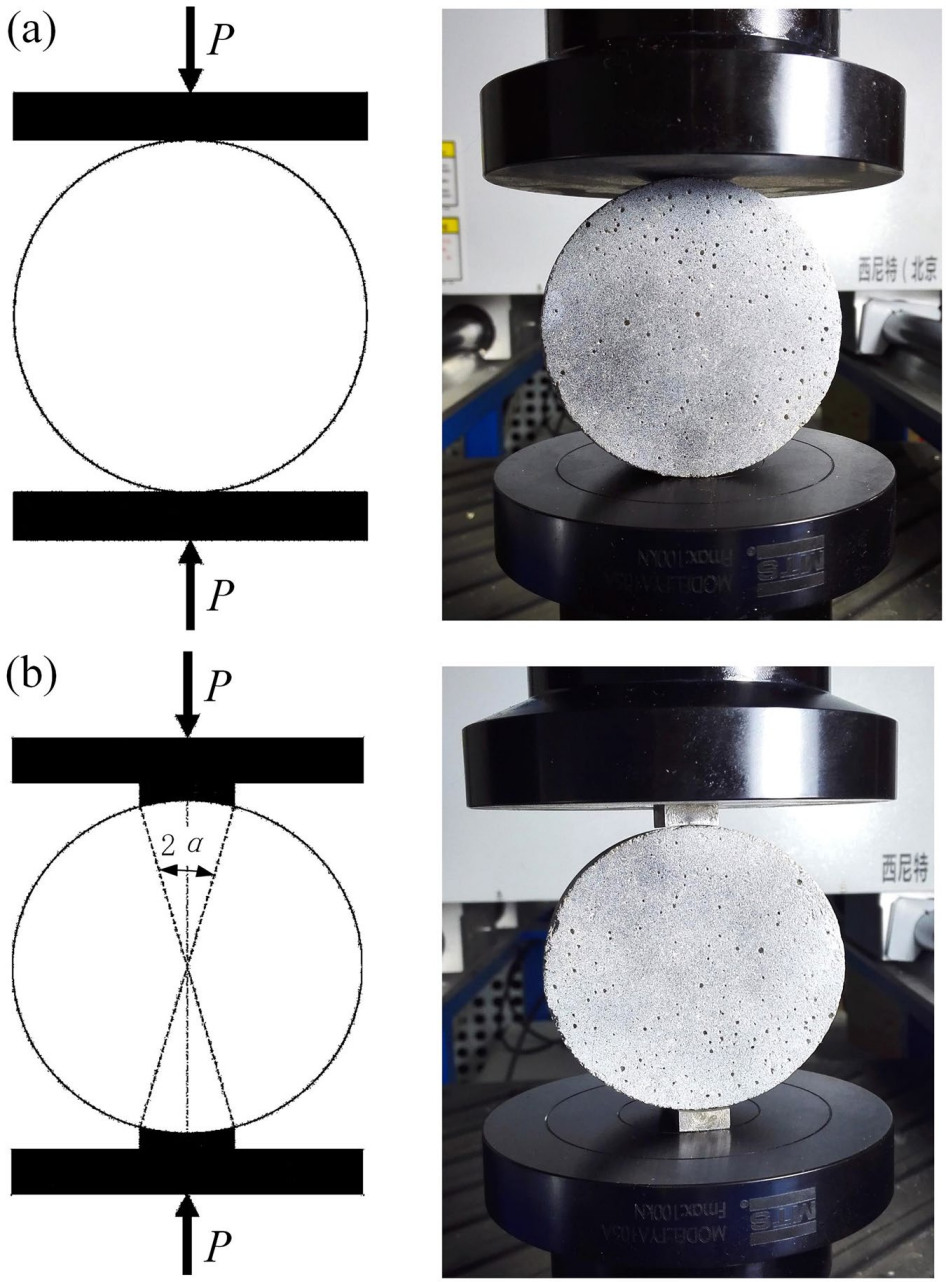
Illustration and photograph of two loading methods: (**a**) concentrated line loading and (**b**) 20° arc loading with bearing strips.

The quasi-static BD tests were also carried out on the MTS testing machine (C45.105) with a load-sensing transducer (LPS.105, 100 kN), according to the standards for evaluation of concrete strengths (GB/T 50081-2002). The loading rate was 0.1 mm/min, and it took several minutes before load reached the maximum value (i.e., specimens split). Here, two different loading methods (concentrated line loading and 20° arc loading with bearing strips), as illustrated in Fig. 2, were adopted to obtain quasi-static tensile strengths of concrete and mortar specimens. In experiments, the two-dimensional digital image correlation (DIC) technique was applied for *in-situ* measurement of displacement and strain in BD specimens with a commercial software (Vic-2D, Vic-Snap, Correlated Solutions, Inc.).

**Figure 3:**
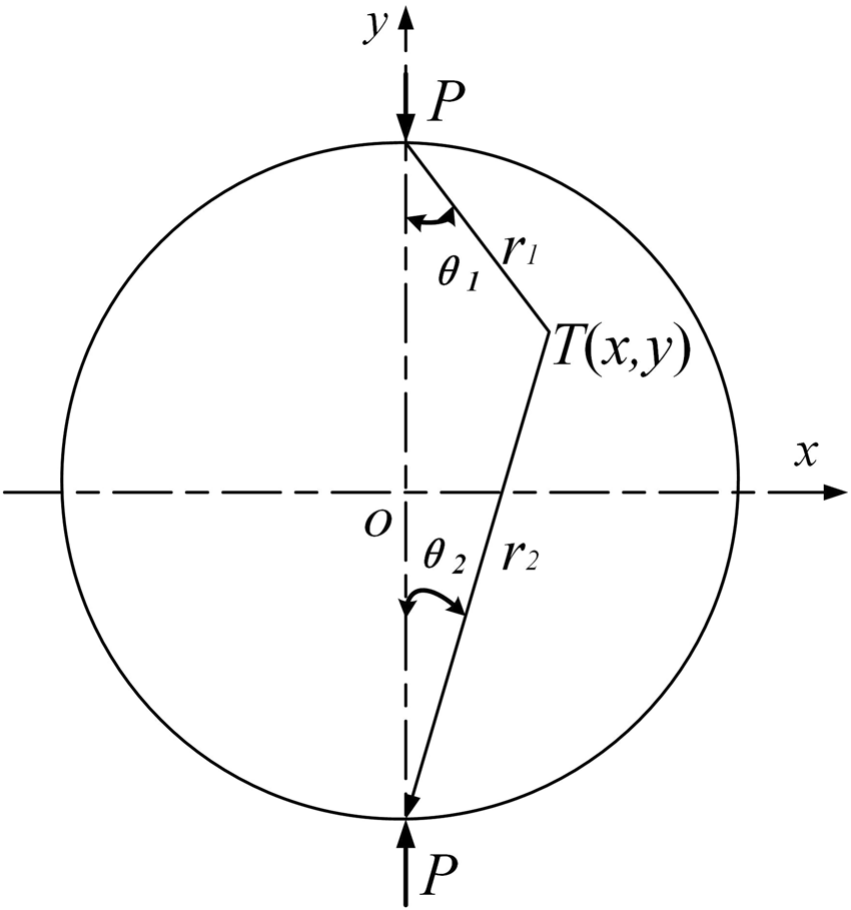
Schematic illustration of the quasi-static splitting test.

Based on the plane-stress hypothesis, when a concentrate line load *P* is applied to a BD specimen, stresses at a point on the specimen, as shown in Fig. 3(a), can be obtained as^26^

2 $${\sigma }_{x}=\frac{2P}{\pi H}(\frac{{\sin }^{2}{\theta }_{1}\,\cos \,{\theta }_{2}}{{r}_{1}}+\frac{{\sin }^{2}{\theta }_{2}\,\cos \,{\theta }_{2}}{{r}_{2}})-\frac{2P}{\pi DH}$$

3 $${\sigma }_{y}=\frac{2P}{\pi H}(\frac{{\cos }^{3}{\theta }_{1}}{{r}_{1}}+\frac{{\cos }^{3}{\theta }_{2}}{{r}_{2}})-\frac{2P}{\pi DH}$$

4 $${\tau }_{xy}=\frac{2P}{\pi H}(\frac{\sin \,{\theta }_{1}{\cos }^{2}{\theta }_{1}}{{r}_{1}}-\frac{\sin \,{\theta }_{2}{\cos }^{2}{\theta }_{2}}{{r}_{2}})$$

where ${\sigma }_{x}$ and ${\sigma }_{y}$ denote stresses at a point *T* in the *x* and *y* directions; *H* and *D* are the thickness and diameter; ${r}_{1}$ and ${r}_{2}$ are the distance from *T* to two loading points; and ${\theta }_{1}$ and ${\theta }_{2}$ are two angles respectively, as illustrated in Fig. 3. At the center point, ${r}_{1}={r}_{2}=0.5D$, the stress state is abbreviated as

5 $${\sigma }_{o,x}=-\frac{2P}{\pi DH}$$

6 $${\sigma }_{o,y}=\frac{6P}{\pi DH}$$

It can be inferred that, at the center point of BD, there is a compressive stress in the *x* direction and a tensile stress in the *y* direction^27^, and the compressive stress ${\sigma }_{o,x}$ is 3 times of tensile stress ${\sigma }_{o,y}$. It is known that compressive strengths of concrete and mortar are about 10–20 times of their tensile strengths. Thus, a vertical crack may firstly occur at the center of BD, and its propagation finally results in failure of the specimen. The tensile strength can be calculated by

7 $${\sigma }_{t}=\frac{2{P}_{t}}{\pi DH}$$

where ${P}_{t}$ is the failure load.

**Figure 4:**
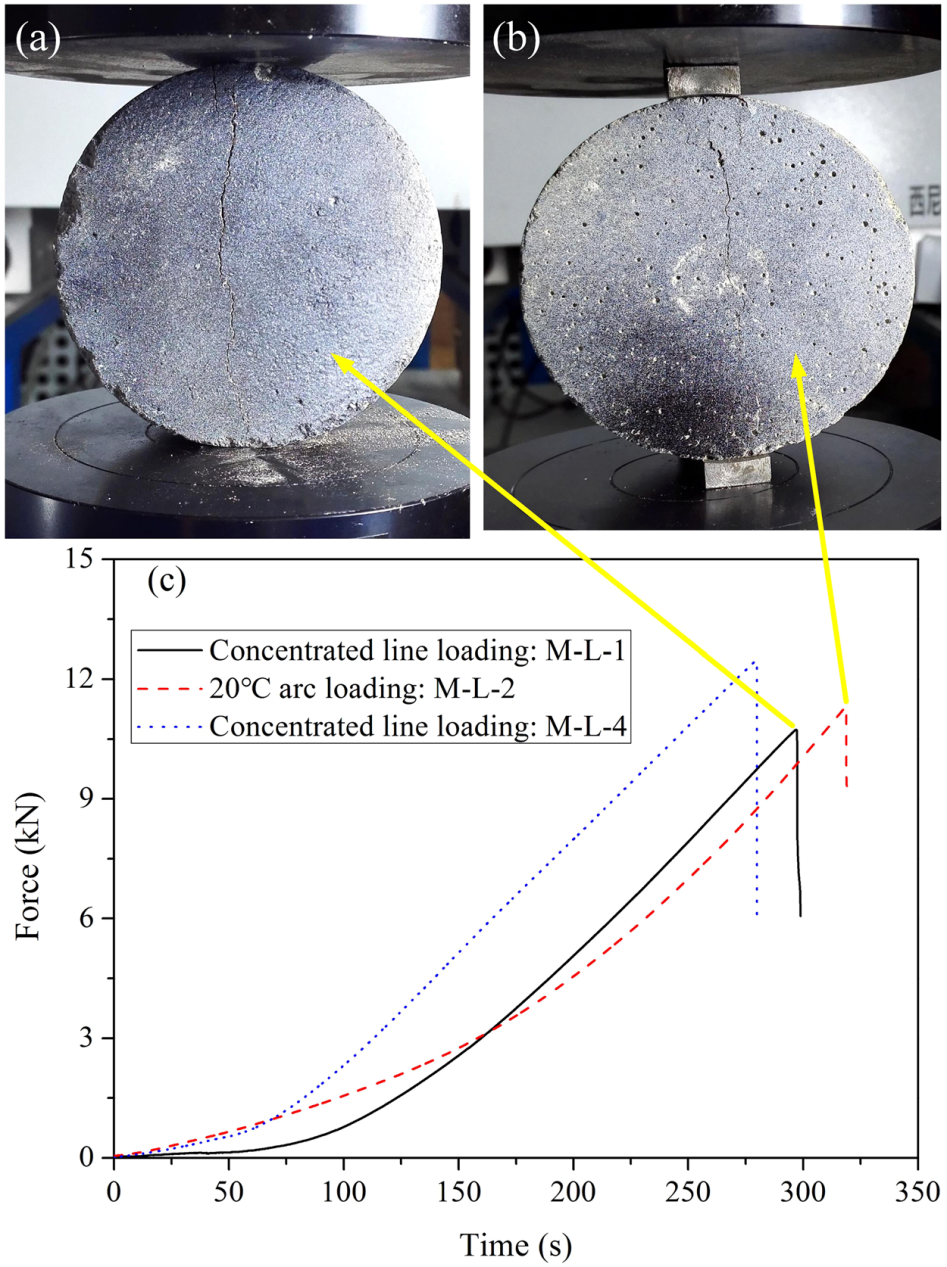
Influence of loading methods: (**a**) the failure mode of M-L-1 with concentrated line loading, and (**b**) the failure mode of M-L-2 with 20° arc loading, and (**c**) their force-time curves.

In splitting tensile tests, there is significant stress concentration near the positions where force is applied through the concentrated line loading, which results in failure of concrete specimens. Thus, to reduce the stress concentration at loading positions, several new loading methods, such as arc loading with bearing strips^28,29^ and flattened BD tests^23,30^, were developed. It is shown that 20° arc loading with bearing strips is an effective method. Here, the effect of two loading methods on the tensile strength was firstly studied with mortar BD specimens. Specimens, M-L-1 and M-L-2 (here, M indicates mortar and L refers to specimens with a thickness of 30 mm), were tested with a load rate of 0.1 mm/min by concentrated line loading and 20° arc loading with bearing strips, respectively. As shown in Fig. 4(a) and (b), specimens M-L-1 and M-L-2 have similar failure modes: a main crack at the center along the loading direction without initiating at the loading point, and only a few small cracks at the edge of load contact. It is obvious to see in Fig. 4(c), that two force-time curves have the same tendency and very similar peak values of 10.73 and 11.30 kN. Similarly, no obvious compressive damage was observed at the loading ends for other three types of specimens with the concentrated line loading method. Thus, it is concluded that the BD specimens are well prepared and the loading methods have little effect on tensile strengths for all the four types of specimens. It is worth noting that, however, the loading methods might still need to be validated in other cases with different properties of concretes. Therefore, for the rest of BD specimens, quasi-static splitting tests were conducted by concentrated line loading with a loading rate of 0.1 mm/min for 20 concrete and 20 mortar specimens, respectively. Results of quasi-static splitting tests are given in Supplementary Table S1.

### Dynamic splitting test

**Figure 5:**
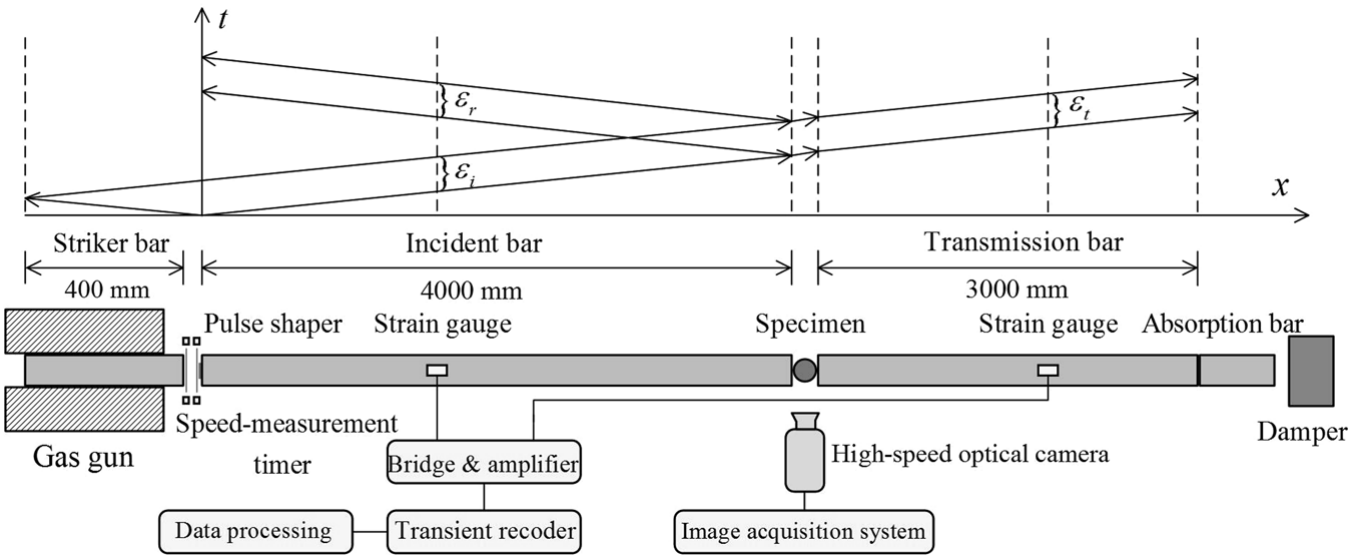
Schematic of a SHPB system and its *x* − *t* diagram of stress wave propagation.

According to the one-dimensional stress wave theory^31^, the stress and particle velocity of a longitudinal stress wave in the SHPB tests can be accurately determined from its associated strain measured by strain gauges, and since both incident and transmission bars are slender rods, it satisfies the plane section assumption. The impact of a striker bar on the free end of an incident bar induces a longitudinal compressive wave propagating in both directions (see Fig. 5). The left-propagating wave is fully released at the free end of the striker bar and forms the trailing end of the incident compressive pulse strain ${\varepsilon }_{i}$. Upon reaching the bar-specimen interface, part of the incident wave is reflected as the reflection wave ${\varepsilon }_{r}$ and the remainder passes through the specimen to the transmitted bar as the transmission wave ${\varepsilon }_{t}$ ^32,33^, as shown in Fig. 5.

The dynamic forces (*P*_1_ and *P*_2_) on incident and transmitted ends of the specimen can be calculated by

8 $${P}_{1}=EA({\varepsilon }_{i}+{\varepsilon }_{r})$$

9 $${P}_{2}=EA{\varepsilon }_{t}$$

10 $$A=\frac{\pi {D}_{0}^{2}}{2}$$

where *E*, *D*_0_ and *A* are the elasticity modulus, diameter and cross-sectional area of two bars, respectively.

In dynamic splitting tests, the stress equilibrium should be ensured at the two ends^34^, which implies that

11 $${P}_{1}={P}_{2}$$

and then, tensile stress at the central point can be obtained by

12 $$\sigma (t)=\frac{{P}_{1}+{P}_{2}}{\pi HD}=\frac{E{{D}_{0}}^{2}}{2HD}({\varepsilon }_{i}(t)+{\varepsilon }_{r}(t))=\frac{E{{D}_{0}}^{2}}{2HD}{\varepsilon }_{t}(t)$$

where ${\varepsilon }_{t}(t)$ is the strain signal of a transmission wave obtained by strain gages. It is obvious that the tensile strength ${\sigma }_{t}$ is the maximum value of tensile stress at the central point.

As splitting is different from the traditional dynamic compressive or tensile test, the strain rate is determined based on the loading rate. Here, loading and strain rates ($\dot{\sigma }$ and $\dot{\varepsilon })$ are defined as

13 $$\dot{\sigma }=\frac{\sigma }{t}$$

14 $$\dot{\varepsilon }=\frac{\dot{\sigma }}{{E}_{0}}$$

where *t* is the time delay between the start of transmitted stress wave and the peak stress, and *E*_0_ is Young’s modulus of concrete or mortar.

**Figure 6:**
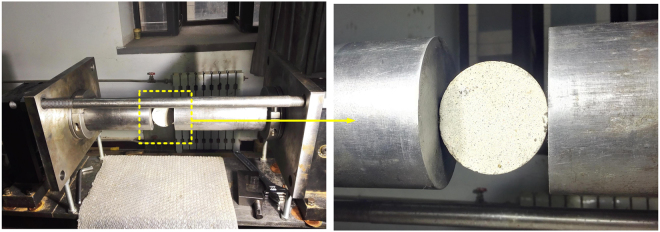
Photograph of a mortar BD specimen in the SHPB system.

**Figure 7:**
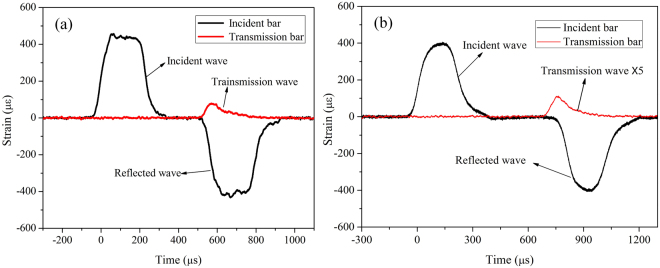
Typical incident, reflected, and transmitted waves in SPHB tests: (**a**) concrete and (**b**) mortar BD specimens, where the transmitted wave signal in the latter is amplified five times for comparison.

As illustrated in Fig. 5, the lengths of strike, incident and transmission bars are 400, 4000 and 3000 mm, respectively, and their diameters are 74 mm. The density of steel bars is 7.80 g·cm^−3^ with an elasticity modulus of 210 GPa. Strain gauges used to measure strain were glued on incident and transmission bars, with 2370 and 1370 mm away from the specimen, respectively. In experiments, an image acquisition system with a high-speed camera was used to record the splitting process. As shown in Fig. 5, to ensure stress equilibrium at two ends of a BD specimen, a copper spacer with a diameter of 20 mm and a thickness of 1 mm was used as the pulse shaper, which can increase the incident stress wave rising or loading time^34^. Thus, it can easily achieve stress equilibrium at the two ends of the specimen. Friction between the specimen and SHPB bars was diminished by using molybdenum disulfide to lubricate contact surfaces, as shown in Fig. 6. Here, it should be mentioned that experimental data obtained from SHPB tests are voltage signals, and after processing, strain and strain rate can be extracted. As the pulse shaping technique has greatly improved the incident wave and increased its rising time, the specimen has enough time to achieve stress uniformity before failure (see Fig. 7). The wave dispersion effect was checked in tests by applying phase shifts to each frequency component of waves^35^. It showed that the influence of wave dispersion on the results was less than 5%. This is mainly attributed to the following reasons: firstly, the ratio of the length to diameter of an incident bar is much more than 20, and this implies that the one-dimensional stress wave theory is well satisfied; secondly, the ratio of the diameter of an incident bar to the wave length is far less than 1, that is, the wave dispersion can be omitted; thirdly, the application of a pulse shaper further reduced the wave dispersion effect. Thus, the wave dispersion effect was not taken into account in experimental analysis. Results of dynamic splitting tests are given in Supplementary Tables S2 and S3.

## Results and Discussion

### Quasi-static splitting

#### Tensile strength

As listed in Supplementary Table S1, the average splitting tensile strengths are 2.25 and 2.73 MPa for 5 concrete specimens with thicknesses of 30 and 55 mm; while their corresponding values for 5 mortar specimens are 3.44 and 3.56 MPa, respectively. Here, the strain rate was about 2.38 × 10^−5^ s^−1^ in tests, which is low enough for us to treat these average values as the static strengths of concrete and mortar. Obviously, the static splitting tensile strength of mortar is higher than that of concrete. During the loading process, micro-cracks occurring at the interface between cement paste and coarse aggregates/sands under tension cause nucleation and propagation of a main crack, and finally result in failure of specimens. In these specimens, cement paste plays an adhesive role that glues sands and coarse aggregates together. Because the interface bonding strength between cement paste and coarse aggregates is weaker than that between cement paste and sands, interface failure of concrete specimens would happen firstly. Here it is worth noting that, concrete specimens were prepared with crushed limestones as coarse aggregates while mortar specimens were made without coarse aggregates. It is the structural difference that causes a higher tensile strength of mortar. As discussed above, the splitting tensile strength is calculated based on the plane-stress state hypothesis. For concrete and mortar specimens with a thickness of 55 mm, the splitting tensile strengths are a little lower than that of specimens with a thickness of 30 mm, which is mainly due to the lower tensile strength of a thicker cylinder calculated based on the plane-stress state hypothesis. In addition, it is worth noting that, the size effect may also result in a lower tensile strength for specimens with a larger size^36,37^.

**Figure 8:**
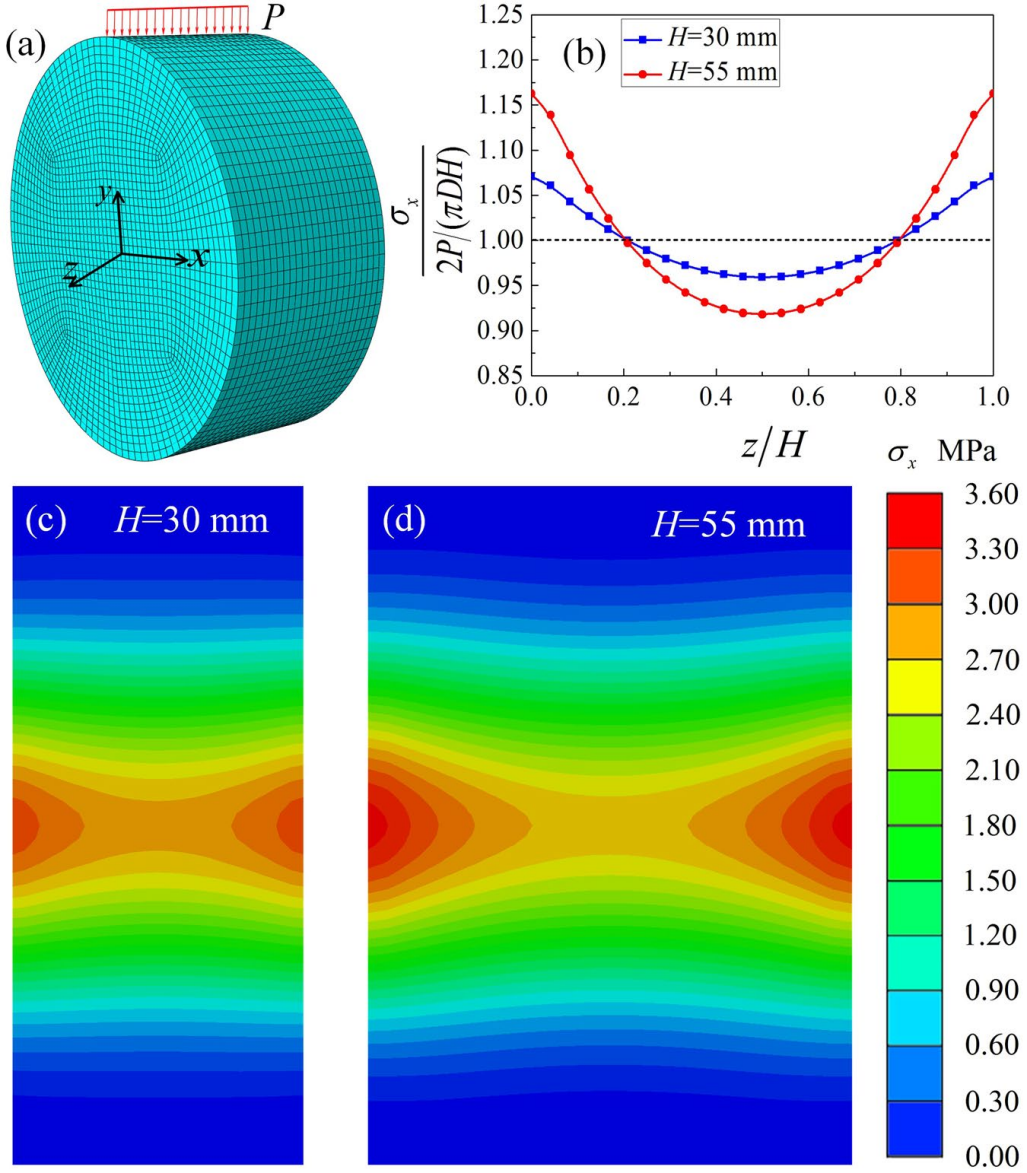
Numerical simulation: (**a**) FEM model, (**b**) dimensionless stresses in x-direction of the points on the axis of symmetry, (**c**,**d**) counter maps of ${\sigma }_{x}$ in x-z plane of specimens with thickness of 30 and 55 mm, respectively.

To further investigate the influence of plane-stress hypothesis on tensile strength, a series of 3D finite element simulations with Abaqus were performed by using two cylindrical models with a diameter *D* = 70 mm and thickness *H* = 30 mm, or *D* = 70 mm and *H* = 55 mm. The material was chosen with a density of 2.40 g·cm^−3^, an elastic modulus of 20 GPa and a Poisson’s ratio of 0.18. To keep the same theoretical maximum tensile strength (3.03 MPa) calculated by Eq. (7), a line load with a value of 10 kN or 18.33 kN was applied on the specimen (see Fig. 8(a)). As shown in Fig. 8(b–d), the maximum tensile stress is at the central point on side surfaces, while the minimum tensile stress is at the central point of a cylinder. That is, cracks may firstly occur on side surfaces and then result in lead to failure of specimens. In addition, the maximum tensile stress on the specimen with a thickness of 55 mm (3.52 MPa) is obvious higher than that on the specimen with a thickness of 30 mm (3.25 MPa). Therefore, the higher tensile stress on side surfaces leads to failure of a thicker specimen that is easier than a thinner one. Thus, it is confirmed that, based on the plane stress hypothesis, the calculation results are prone to a lower tensile strength. Furthermore, it is also shown that the specimen is not under a strict plane stress state. However, it is appropriate to calculate the tensile strength with the plane-stress hypothesis because the error is less than 7% for a specimen with a thickness of 30 mm.

#### Failure modes

**Figure 9:**
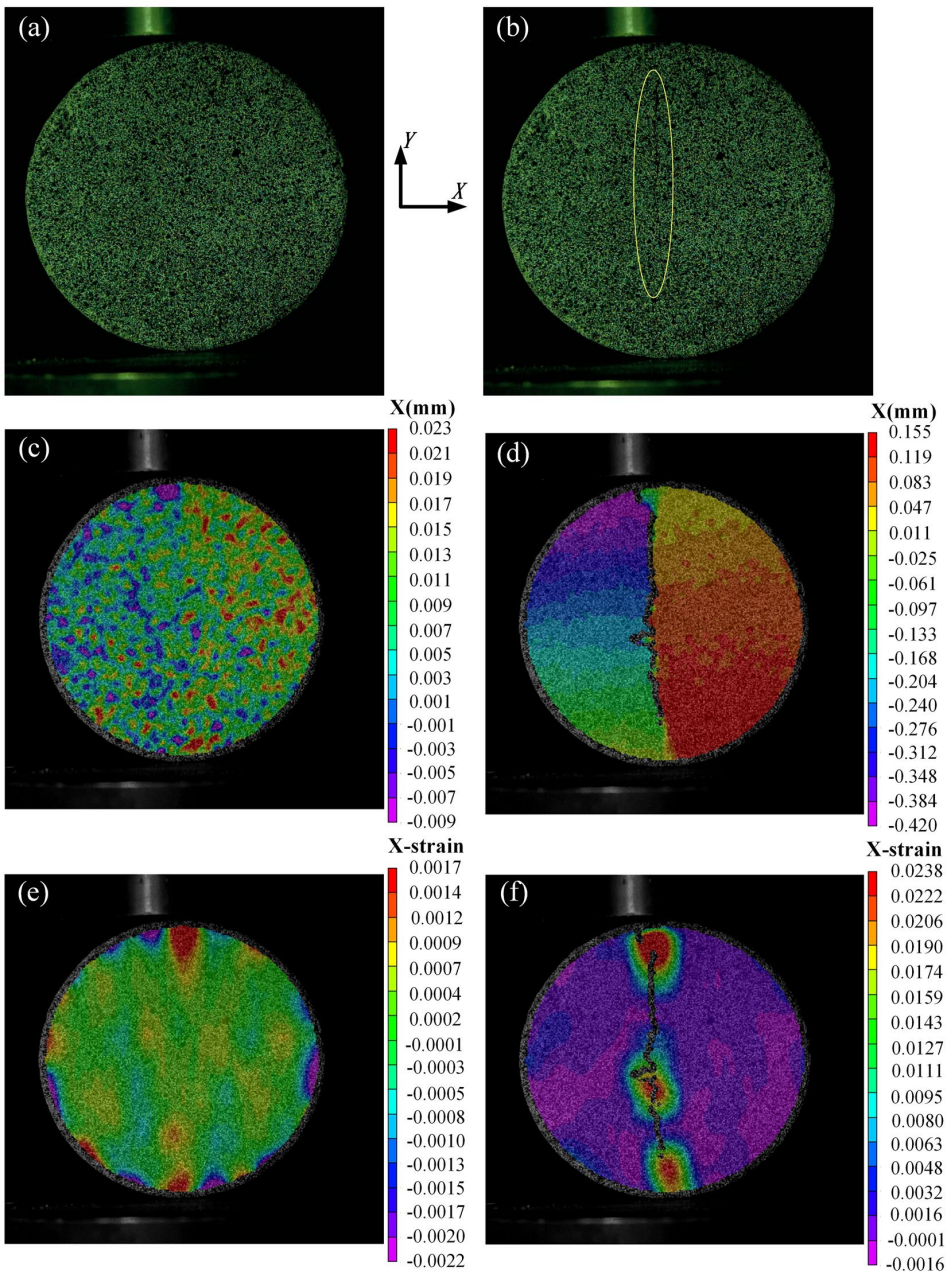
Failure modes of specimen M-L-4: (**a**,**c**,**e**) are morphology, displacement and strain maps perpendicular to the loading direction before failure (*t* = 279.60 s); (**b**,**d**,**f**) are their corresponding maps after failure (*t* = 279.65 s).

**Figure 10:**
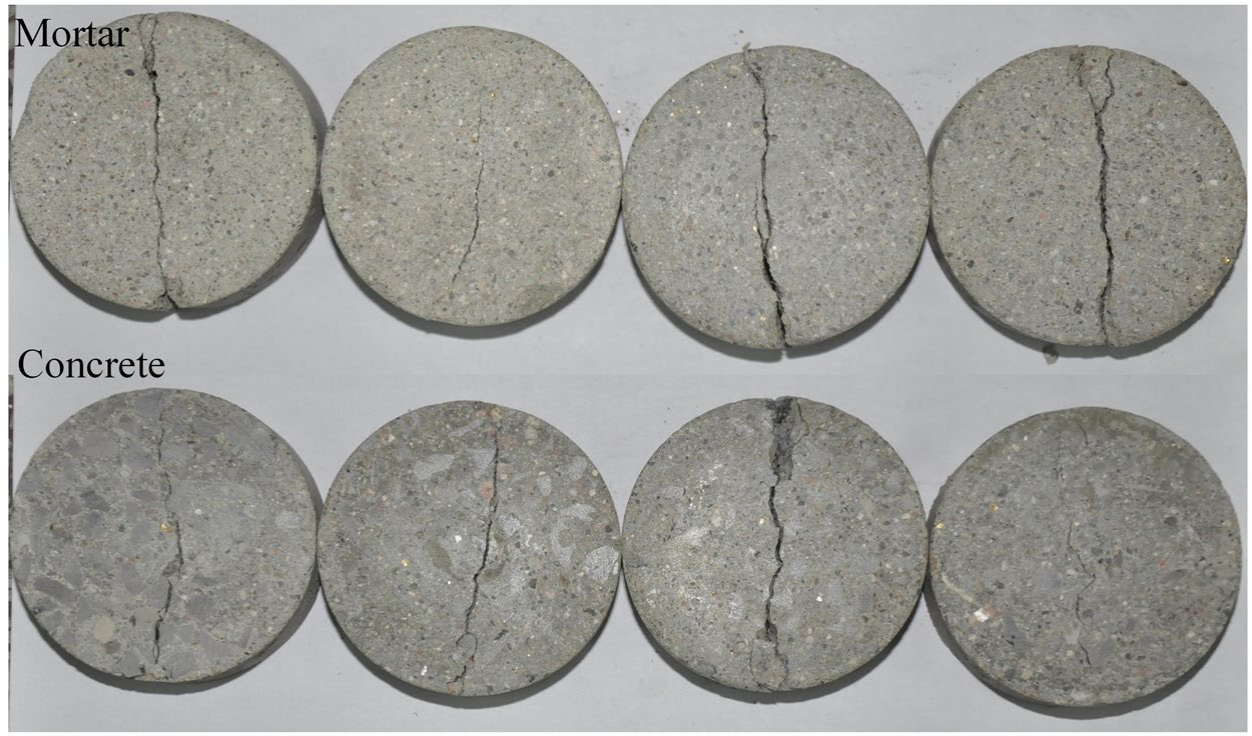
Failure modes of mortar and concrete BD specimens under quasi-static splitting tests with a loading rate of 0.1 mm/min.

As splitting tests are based on the plane-stress state hypothesis, the two-dimensional DIC technique fully meets requirement for *in-situ* measurements, and its application is detailed discussed by using the mortar specimen M-L-4 with a concentrated line loading at 0.1 mm/min for example. As shown in Fig. 4, when loading lasts for 279.65 s, force reaches a maximum value of 12.46 kN, and then drops down quickly due to failure of the specimen. The DIC camera can capture as many as 80 frames per second, and 20 were recorded in quasi-static splitting tests. As observed in Fig. 9, a main crack in the center nucleates along the loading direction in a short time (see Fig. 9(a) and (b), where the time difference is 0.05 s). Further, except for the main crack in the center, no small cracks are observed on the surface of BD specimens. Before the main crack appears, displacement and strain perpendicular to the loading direction are almost uniformly distributed in mortar specimens (see Fig. 9(c)); but after that, displacements in top left and bottom right corners near the main crack are obviously higher than other areas (see Fig. 9(d)). Such a displacement distribution implies that there is a slight rotation of the BD specimen, which can be confirmed by the video record. Similarly, strain is only observed near the loading point on surface before there is a main crack, and the BD specimen is under a relatively low strain level, as shown in Fig. 9(e). As soon as the crack forms, the strain level on its sides suddenly increases by an order of magnitude, as shown in Fig. 9(f). It is obvious that the largest tensile strain on mortar specimens is 0.02 in x-direction (along the diametral loading direction), It can be further inferred that when the tensile strain reaches 0.02, the macrocrack occurs and finally leads to the fracture of the mortar specimen, which is very close to the value used in erosion simulations^19,25^. That is, the DIC technique is effective in investigating the failure process of concrete-like materials. As collected in Fig. 10, it is found that, for both mortar and concrete BD specimens, there is only a main crack in the center along the loading direction, without crushing zones formed at loading ends. That is, there is no obvious compressive stress concentration, and specimens fracture were due to compressive damage.

### Dynamic splitting

#### Dynamic tensile strength

To obtain the stress-time relationship, the time of incident and reflected stresses is recorded when the incident bar impacts a specimen; similarly, the time of transmission stress starts when a specimen contacts with the transmission bar. Based on these stress-time curves, the dynamic force on the both ends of the BD specimen can be obtained. As a result, the dynamic stress equilibrium at the center of the BD specimen can be guaranteed via the force balance on its boundaries (Supplementary Fig. S3).

**Figure 11:**
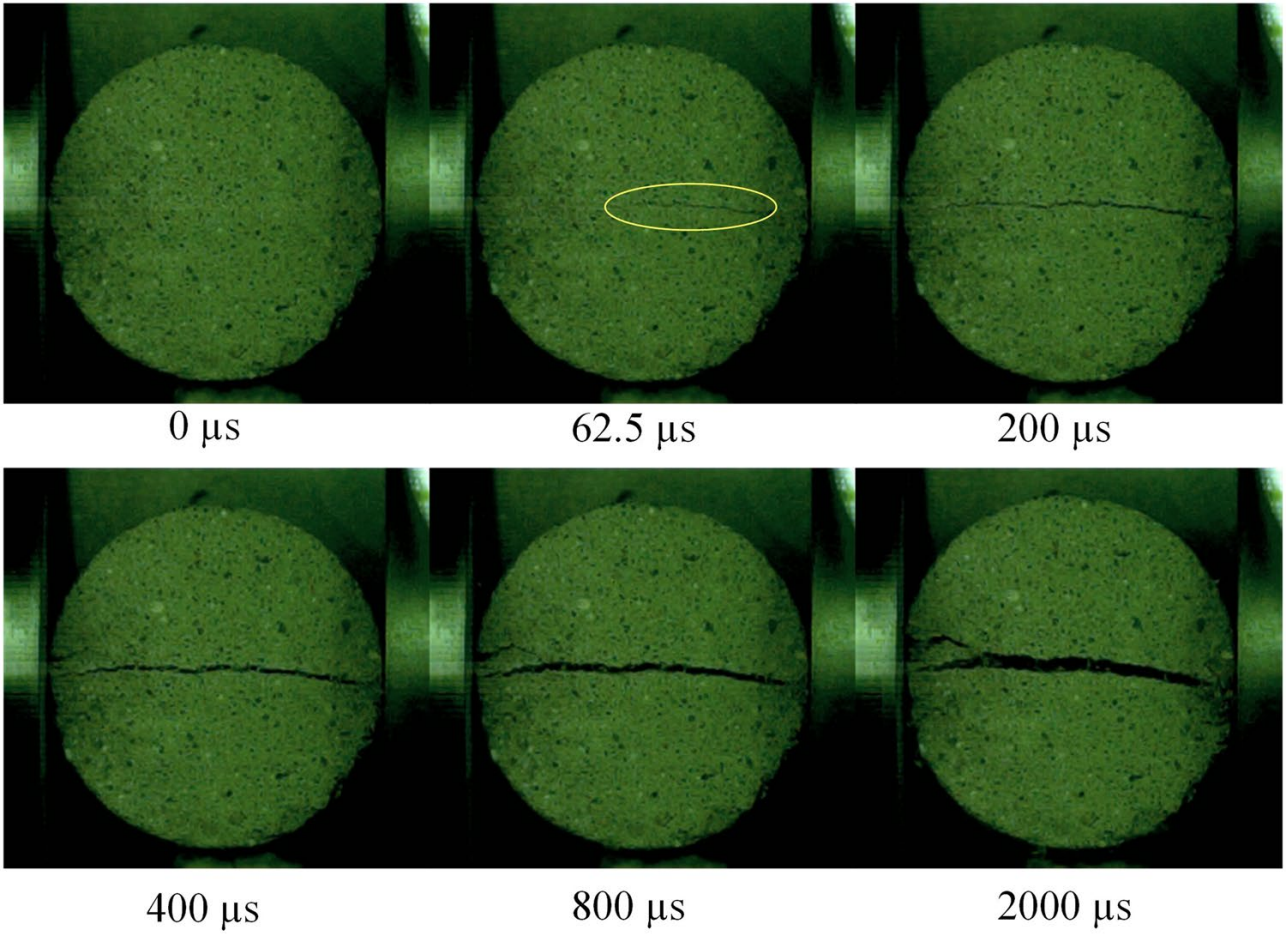
High speed camera snapshots during the failure process of mortar BD specimen M-H-11.

**Figure 12:**
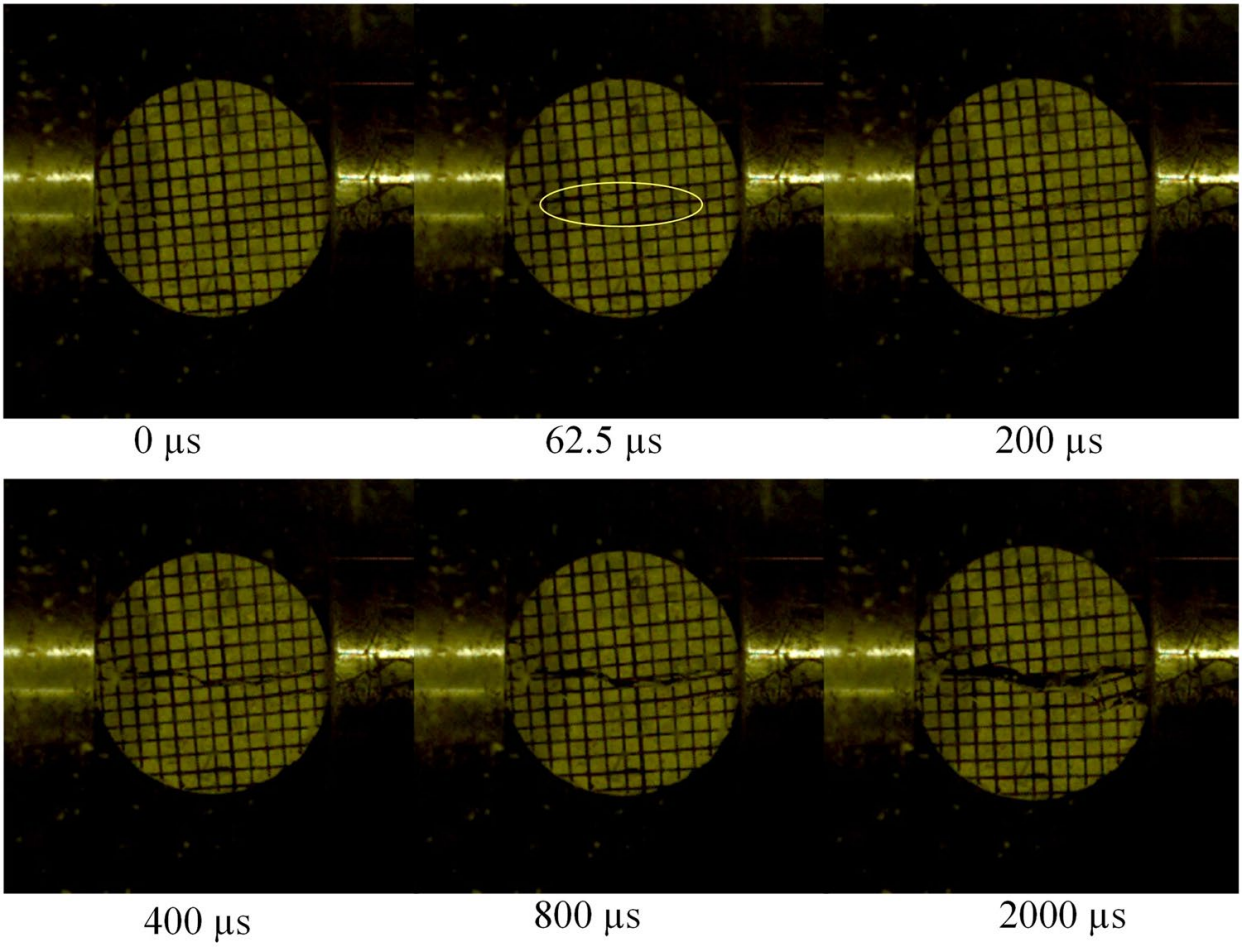
High speed camera snapshots during the failure process of concrete BD specimen C-L-22.

The start-split location and crack propagation are two important indicators for the BD tests of concrete. A test is considered as valid only when the start-split location is at the center and cracks propagate along the loading diameter direction. As shown in Supplementary Fig. S3, dynamic force on the end surface reaches the peak value at about 60 μs and then drops down quickly, which means that the BD specimen fails. As overserved from high speed camera snapshots obtained by splitting experiments with a relative low gas pressure (see Figs 11 and 12), it is obvious that, for both mortar and concrete, a single main crack occurring in their centers at 62.5 μs propagates along the loading direction, and then the BD specimens are separated into two halves. Based on the force balance, dynamic tensile strengths of BD specimens can be calculated by Eq. (12). The results of the tensile strengths and DIF for concrete and mortar specimens were listed in Supplementary Tables S2 and S3, respectively. It is obvious that the dynamic tensile strengths of concrete and mortar specimens increase with the increase of stain rates, and their DIFs have the same trend.

#### Failure modes

**Figure 13:**
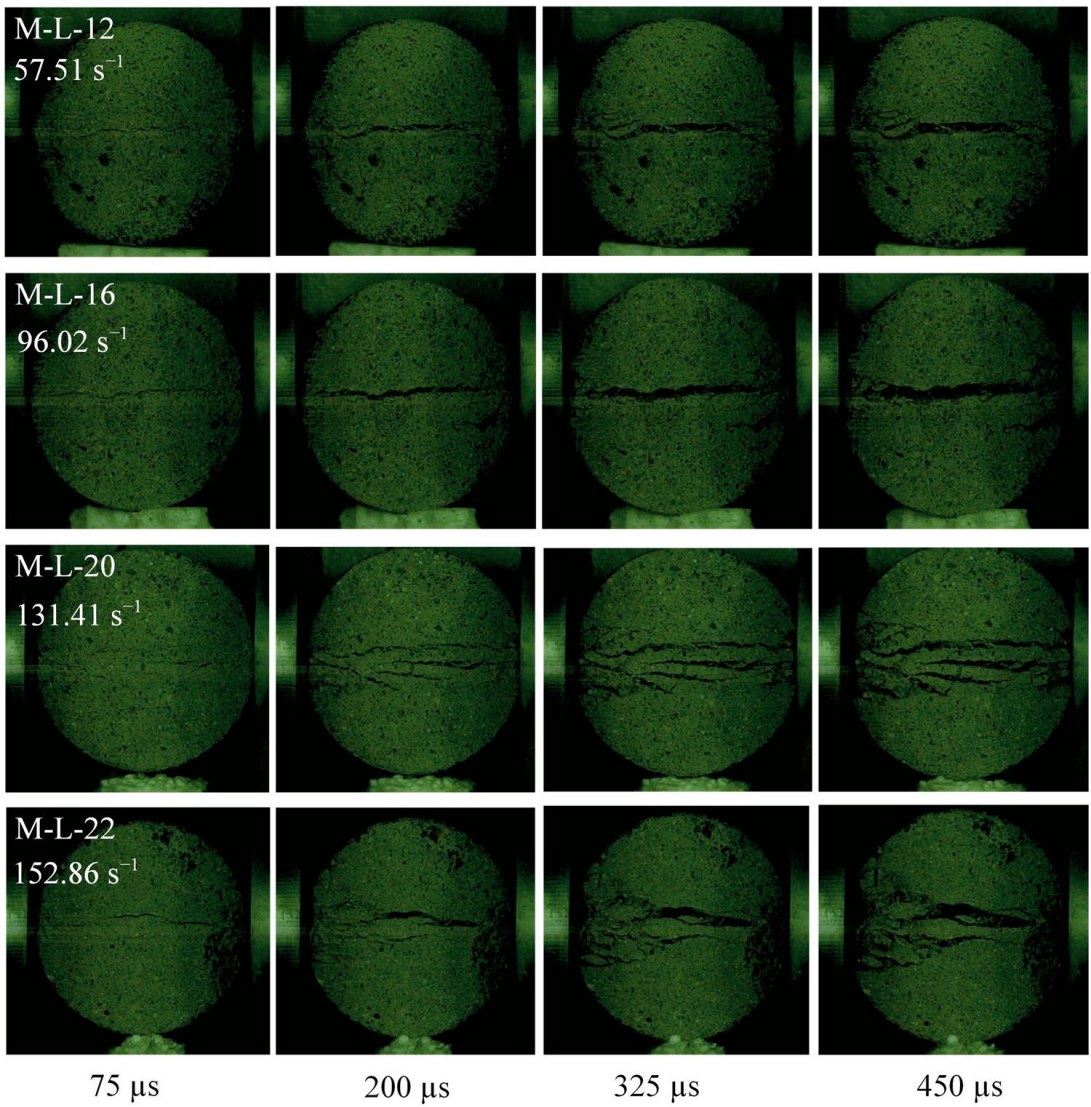
Failure process in mortar BD specimens with a thickness of 30 mm under different strain rates.

**Figure 14:**
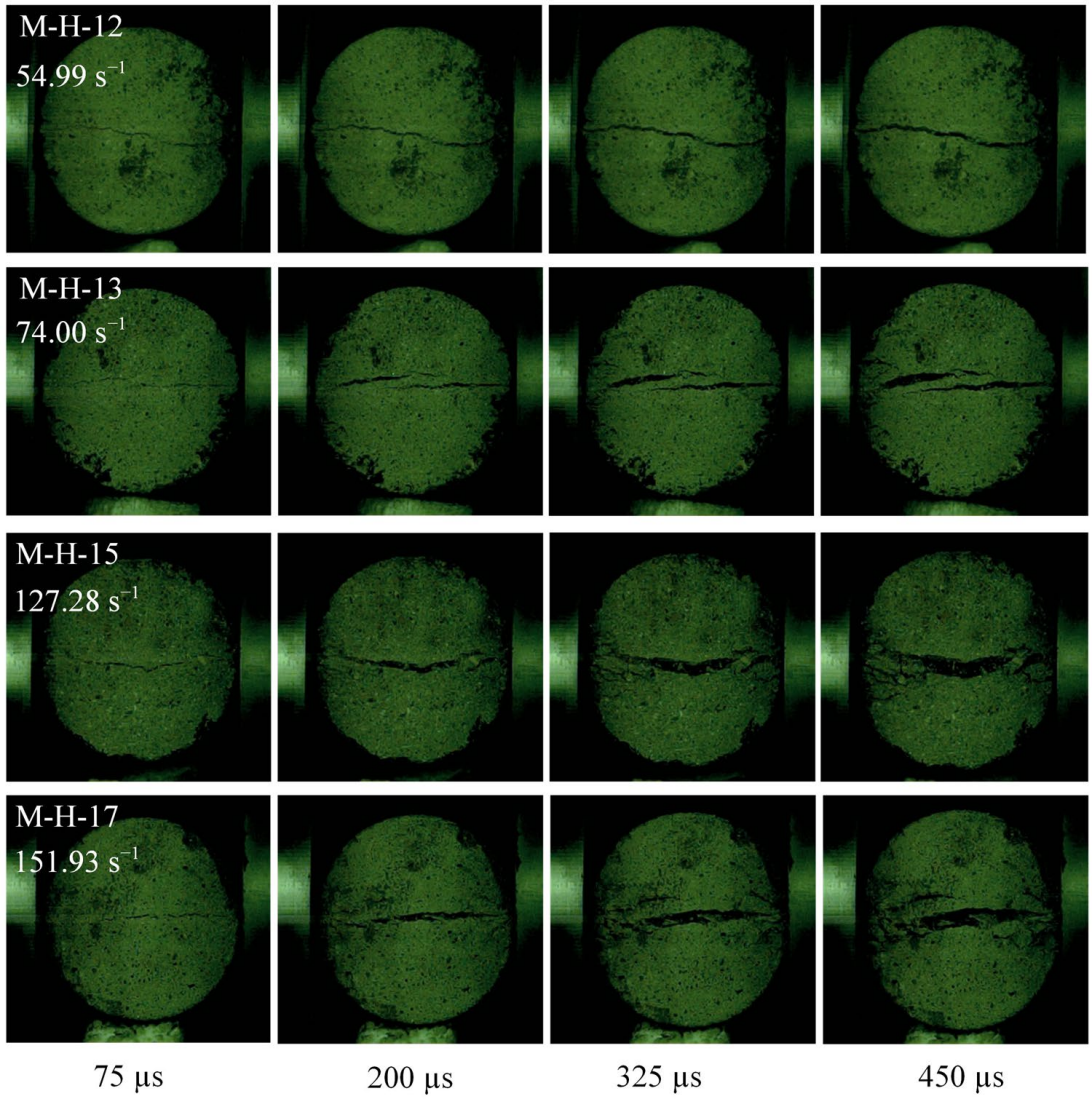
Failure process in mortar BD specimens with a thickness of 55 mm under different strain rates.

To highlight the effect of strain rate on failure modes, the failure process of mortar BD specimens was carefully observed by high speed camera. Because four different gas pressures were used in splitting tests, the strain rate significantly concentrates in four ranges. For mortar BD specimens with a thickness of 30 mm, four different strain rates are 57.51, 96.02, 131.41, and 152.86 s^−1^. As seen in Fig. 13, a single crack failure exhibits in mortar BD specimens under strain rates of 57.51 and 96.02 s^−1^, since there is only a main crack that forms and splits them into two halves. For the BD specimen tested with a strain rate of 131.41 s^−1^, multiple cracks are observed after a main crack. When the strain rate reaches 152.86 s^−1^, failure pattern changes to fragment with a wedge fragment region. The failure patterns might be different for mortar BD specimens with different thicknesses. Thus, mortar BD specimens with a thickness of 55 mm were further investigated and compared under four different strain rates of 54.99, 74.00, 127.28, and 151.93 s^−1^ (see Fig. 14). It is shown that these specimens exhibit a single crack (M-H-12), multiple cracks (M-L-13) and fragment failure (M-H-15 and M-L-17), respectively. Similar phenomena were also observed in refs^38,39^. Here, it is worth emphasizing that, there was a center crack formed before multiple cracks or wedge fragment. It implies that failure of BD specimens is indeed due to tensile stress at the center in dynamic splitting tests. Thus, the dynamic tensile strength of BD specimens can still be calculated by the maximum force on their end surfaces.

### Dynamic increase factor

**Figure 15:**
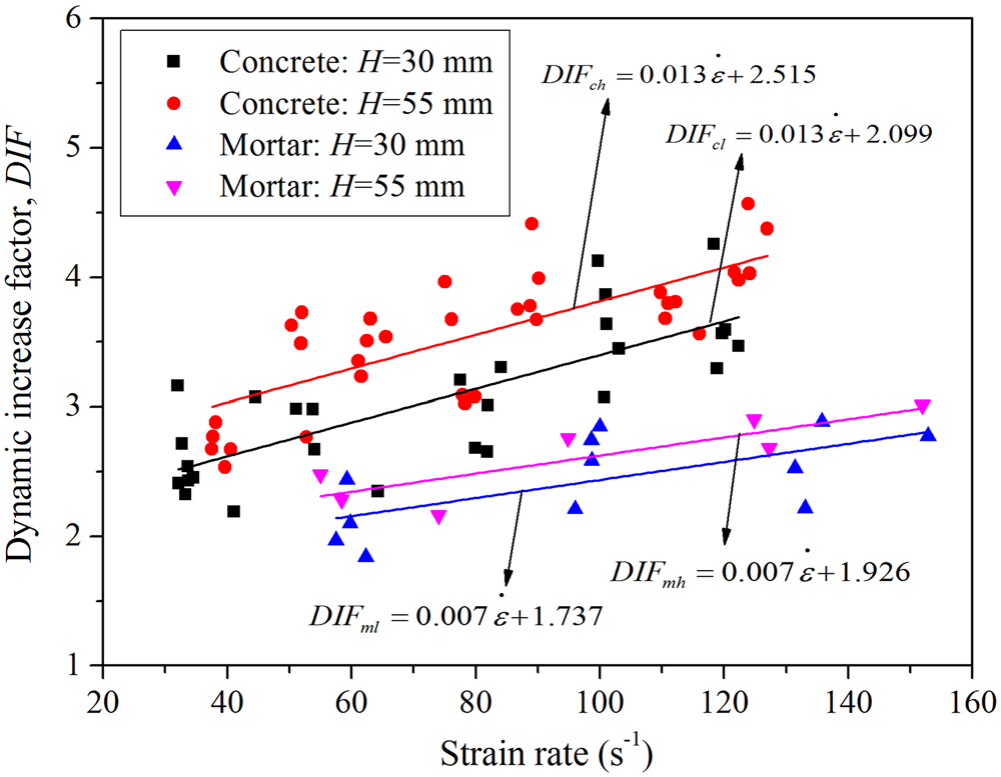
Relationships between the DIF and strain rate for concrete and mortar BD specimens.

The DIFs for concrete and mortar BD specimens were calculated based on quasi-static and dynamic tensile strengths obtained from splitting tests (see Supplementary Tables S2 and S3. To evaluate the strain rate effect on DIF, a linear fitting algorithm was used, and the fitted formulas were given in Fig. 15. For both mortar and concrete, the fitted straight lines for BD specimens with a thickness of 30 mm are parallel to that of specimens with a thickness of 55 mm, as shown in Fig. 15. To quantitatively describe the variability and scatter of DIFs, the relative deviation *δ* can be calculated by

15 $$\delta =\frac{1}{n}\sum _{i=1}^{n}\frac{|DI{F}_{i}-DI{{F}_{i}}^{^{\prime} }|}{DI{{F}_{i}}^{^{\prime} }}\times 100 \% $$

where $DI{F}_{i}$ refers to one obtained from dynamic splitting tests, and $DI{{F}_{i}}^{^{\prime} }$ are the corresponding value calculated by the fitted equation with the same strain rate. As results, their relative deviations are 9.12%, 8.10%, 9.54% and 5.33% for $DI{F}_{ch}$, $DI{F}_{cl}$, $DI{F}_{mh}$ and $DI{F}_{ml}$, respectively.

The difference of DIFs for specimens with different thicknesses is caused by the inertial and size effects. The slopes of linear fitting equations are quite close, which reflect the real strain effect on dynamic tensile strength. Further investigation shows that the DIF for concrete is higher than that of mortar. As discussed above, the average quasi-static tensile strength of mortar is much higher than that of concrete, which directly causes a lower DIF of mortar. This agrees with the numerical results of DIFs under dynamic compression^15^, in which concrete and mortar were simulated with mesoscale and homogeneous models, respectively.

As discussed above, the DIF of concrete is obviously higher than that of mortar, thus, DIFs of these two materials should be separately calculated. Taking the lower inertial, size effects and the plane stress hypothesis in to account, the linear fitting equations for specimens with a thickness of 30 mm are recommended in calculation of DIFs. That is, DIFs for concrete and mortar materials can be obtained by

16 $$DI{F}_{c}=0.013\dot{\varepsilon }+2.099$$

17 $$DI{F}_{m}=0.007\dot{\varepsilon }+1.737$$

Here, it is worth noting that there are few researches on the relationship between the DIF and strain rate of concrete-like materials in the range of 1–200 s^−1^. Thus, as a valuable attempt, the formulas proposed in Eqs. (16) and (17) can be conveniently used in applications to calculate the dynamic tensile strength of concrete and mortar materials.

To the best of our knowledge, there are still no studies on the effects of factors such as types and sizes of aggregates on DIF. With the size increase of aggregates, the density of concrete increases, and a larger specimen is necessary to ensure its isotropy, both of which will have obvious effect on the DIF. In addition, these factors may increase the inertial effect of a specimen in dynamic splitting tests, and lead to a higher dynamic tensile strength and DIF. Thus, the influence of strength on DIF needs further investigation.

## Conclusions

In this paper, the tensile strength and failure pattern of concrete and mortar were investigated under quasi-static and dynamic loadings based on splitting BD tests. The main conclusions can be drawn as follows:

1. The quasi-static tensile strength of mortar is higher than that of concrete because coarse aggregates weaken the interface bonding strength. Simulation results show that the maximum tensile stress is at the central point on side surfaces, and the minimum tensile stress is at the central point of a cylinder. Thus, it is confirmed that the calculation based on plan stress hypothesis leads to a lower tensile strength.
2. A macrocrack finally leads to fracture of a mortar specimen when tensile strain reaches a certain value. The strain maps obtained with a DIC technique show that macrocracks nucleate between 0.002 and 0.02.
3. With the increase of strain rate, the dynamic tensile strength of concrete and mortar BD specimens significantly increases, and their failure patterns change form a single crack to multiple cracks and even fragment. For three failure modes, there is a center crack before multiple cracks and wedge fragment, which implies that failure of BD specimens is indeed caused by the tensile stress at the center in dynamic splitting tests.
4. Two fitted equations are proposed for the strain rate in the range of 1–200 s^−1^. They can be conveniently applied to determine the dynamic tensile strengths and to calculate the DIFs of concrete and mortar materials.

## Electronic supplementary material

Supplementary information
