# Author Correction: Quasi-static and dynamic experimental studies on the tensile strength and failure pattern of concrete and mortar discs

**DOI:** 10.1038/s41598-022-14641-9

**Published:** 2022-07-01

**Authors:** Xiaochao Jin, Cheng Hou, Xueling Fan, Chunsheng Lu, Huawei Yang, Xuefeng Shu, Zhihua Wang

**Affiliations:** 1 State Key Laboratory for Strength and Vibration of Mechanical Structures, School of Aerospace Engineering, Xi’an Jiaotong University, Xi’an, 710049 China; 2 Department of Mechanical Engineering, Curtin University, Perth, Western Australia 6845 Australia; 3 Shanxi Key Laboratory of Material Strength and Structural Impact, Taiyuan University of Technology, Taiyuan, 030024 China

Correction to: *Scientific Reports* https://doi.org/10.1038/s41598-017-15700-2, published online 10 November 2017

The original version of this Article contained errors.

The article contained typos in two equations. The Eqs. (2) and (10) should read

2 $${\sigma }_{x}=\frac{2P}{\pi H} \left (\frac{{\mathit{sin}}^{2}{\theta }_{1}\mathit{cos}{\theta }_{1}}{{r}_{1}}+\frac{{\mathit{sin}}^{2}{\theta }_{2}\mathit{cos}{\theta }_{2}}{{r}_{2}}\right)-\frac{2P}{\pi DH}$$

10 $$A=\frac{\pi {D}_{0}^{2}}{4}$$

Additionally, there were errors in the average quasi-static splitting strengths of concrete and mortar.

“As listed in Supplementary Table S1, the average splitting tensile strengths are 2.25 and 2.73 MPa for 5 concrete specimens with thicknesses of 30 and 55 mm; while their corresponding values for 5 mortar specimens are 3.44 and 3.56 MPa, respectively.”

should read:

“As listed in Supplementary Table S1, the average splitting tensile strengths are 2.64 and 2.60 MPa for 10 concrete specimens with thicknesses of 30 and 55 mm; while their corresponding values for 10 mortar specimens are 3.47 and 3.35 MPa, respectively.”

Finally, in Figure 7(a), the transmission wave signal was amplified twice without correct marking. The corrected Figure 7(a) appears below.

These errors do not affect the conclusions of the Article.

**Figure 7:**
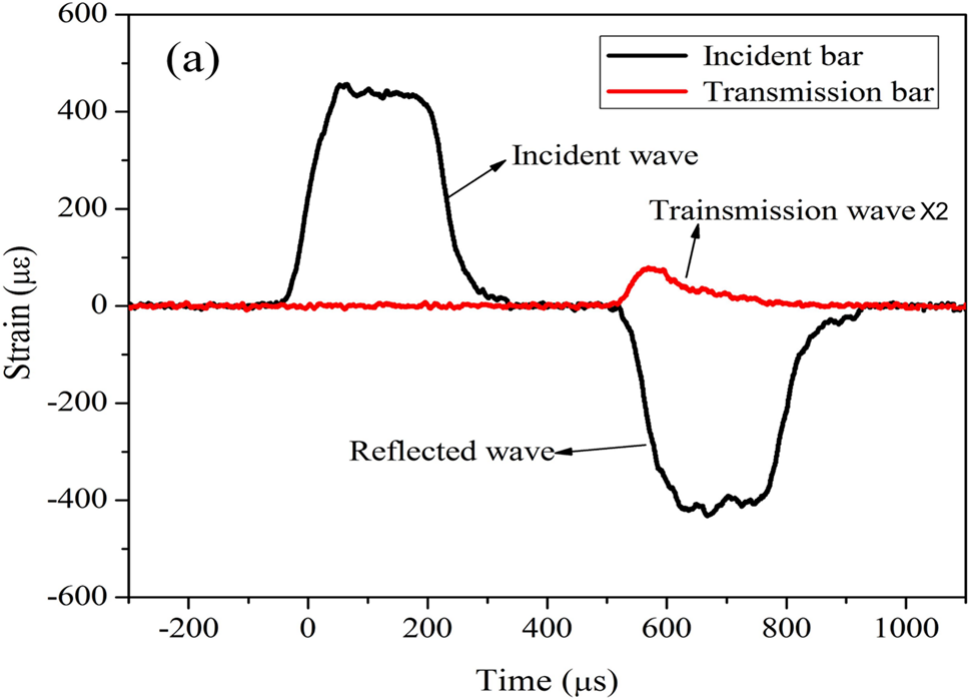
Typical incident, reflected, and transmitted waves in SPHB tests: (**a**) concrete BD specimens, where the transmitted wave signal in the latter is amplified twice to aid observation.

