# Supplementary material for: Quasi-static and dynamic experimental studies on the tensile strength and failure pattern of concrete and mortar discs
Source: Sci Rep. 2017 Nov 10;7:15305. doi: 10.1038/s41598-017-15700-2 (PMC5681700; doi:10.1038/s41598-017-15700-2)
Supplement: Supplementary file 1 — Supplementary information [file 41598_2017_15700_MOESM1_ESM.doc]

**Quasi-static and dynamic experimental studies on the tensile strength and failure pattern of concrete and mortar discs**

Xiaochao Jin^1^, Cheng Hou^1^, Xueling Fan 1,[[1]](#footnote-2)*, Chunsheng Lu^2^, Huawei Yang^3^, Xuefeng Shu^3^, Zhihua Wang^3^

^1^ State Key Laboratory for Strength and Vibration of Mechanical Structures, School of Aerospace Engineering, Xi’an Jiaotong University, Xi’an 710049, China

^2^ Department of Mechanical Engineering, Curtin University, Perth, Western Australia 6845, Australia

^3^ Shanxi Key Laboratory of Material Strength and Structural Impact, Taiyuan University of Technology, Taiyuan 030024, China

*Corresponding Author:* [fanxueling@mail.xjtu.edu](mailto:fanxueling@mail.xjtu.edu).

*Tel.:* +86-29-82667864; Fax: +86-29-82669044

*Postal address:* No.28, Xianning West Road, Xi’an, Shaanxi, 710049,

School of Aerospace, Xi’an Jiaotong University, China

**Supplementary Figure S1**


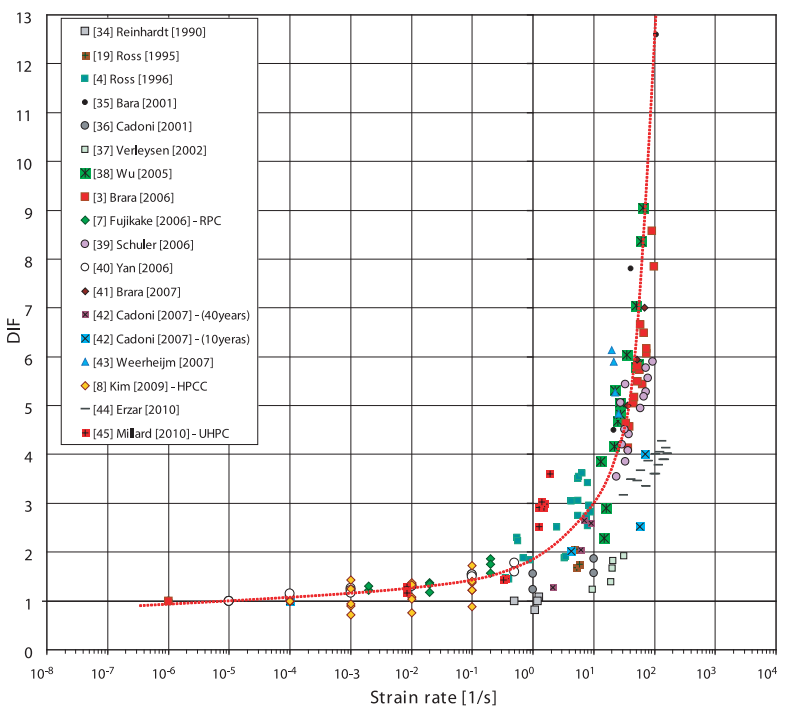


Figure S1. Strain-rate influence on the tensile strength of concrete [7].

**Supplementary Figure S2**


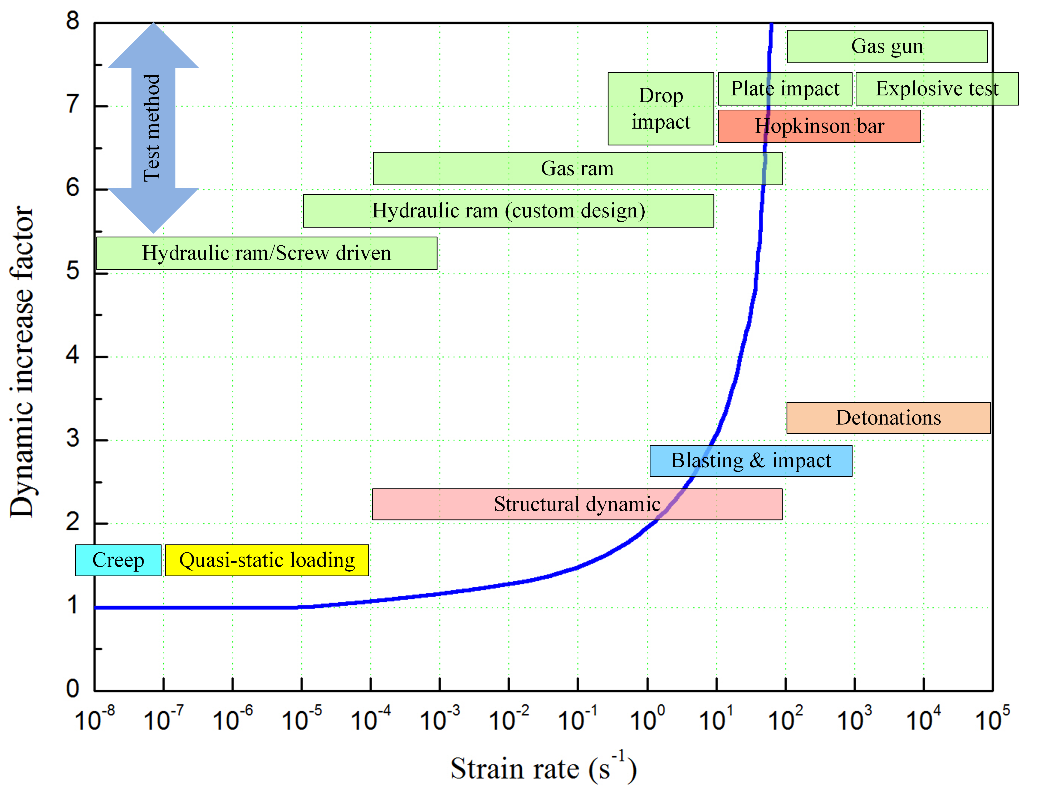


Figure S2. Classification of dynamic problems and testing methods over a wide range of strain rates (adapted from Ref. [27]).

**Supplementary Figure S3**


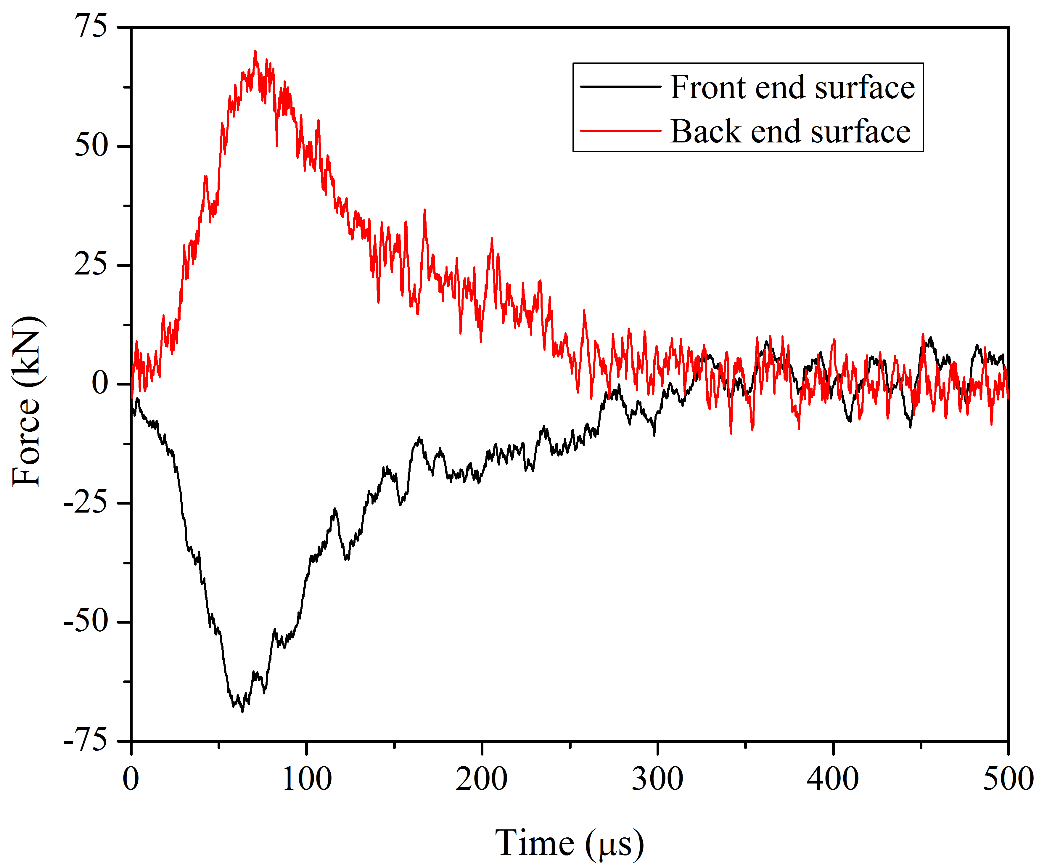


Figure S3. Dynamic force versus time in a typical dynamic BD test.

**Supplementary Table S1**

Table S1 Results of quasi-static splitting tests, in which C and M represent concrete and mortar, and L and H refer to specimens with a thickness of 30 and 55 mm, respectively.

| **Specimen** | **Failure load (kN)** | **Tensile strength (MPa)** | **Specimen** | **Failure load (kN)** | **Tensile strength (MPa)** |
| --- | --- | --- | --- | --- | --- |
| C-L-1 | 9.50 | 2.88 | M-L-1 | 10.73 | 3.25 |
| C-L-2 | 7.53 | 2.28 | M-L-2 | 11.30 | 3.43 |
| C-L-3 | 8.64 | 2.62 | M-L-3 | 11.22 | 3.40 |
| C-L-4 | 8.91 | 2.70 | M-L-4 | 12.46 | 3.78 |
| C-L-5 | 9.86 | 2.99 | M-L-5 | 11.12 | 3.37 |
| C-L-6 | 8.36 | 2.53 | M-L-6 | 11.37 | 3.45 |
| C-L-7 | 9.34 | 2.83 | M-L-7 | 11.37 | 3.45 |
| C-L-8 | 7.89 | 2.39 | M-L-8 | 10.92 | 3.31 |
| C-L-9 | 8.24 | 2.50 | M-L-9 | 12.05 | 3.65 |
| C-L-10 | 8.82 | 2.67 | M-L-10 | 11.86 | 3.60 |
| Average value | 8.71 | 2.64 | Average value | 11.44 | 3.47 |
| C-H-1 | 16.22 | 2.68 | M-H-1 | 21.74 | 3.59 |
| C-H-2 | 17.49 | 2.89 | M-H-2 | 20.89 | 3.45 |
| C-H-3 | 15.44 | 2.55 | M-H-3 | 20.74 | 3.43 |
| C-H-4 | 15.67 | 2.59 | M-H-4 | 21.80 | 3.60 |
| C-H-5 | 15.29 | 2.53 | M-H-5 | 20.63 | 3.41 |
| C-H-6 | 15.64 | 2.59 | M-H-6 | 19.54 | 3.23 |
| C-H-7 | 16.05 | 2.65 | M-H-7 | 19.58 | 3.24 |
| C-H-8 | 15.42 | 2.55 | M-H-8 | 20.26 | 3.35 |
| C-H-9 | 14.52 | 2.40 | M-H-9 | 18.25 | 3.02 |
| C-H-10 | 15.34 | 2.54 | M-H-10 | 19.14 | 3.16 |
| Average value | 15.71 | 2.60 | Average value | 20.26 | 3.35 |

**Supplementary Table S2**

Table S2. Results of dynamic splitting tests with BDs, where C represents concrete, and L and H refer to specimens with a thickness of 30 and 55 mm, respectively.

| **Specimen** | **Gas pressure (MPa)** | **Strain rate (s^−1^)** | **Tensile strength (MPa)** | **DIF** |
| --- | --- | --- | --- | --- |
| C-L-11 | 0.08 | 32.05 | 8.34 | 3.16 |
| C-L-12 | 0.08 | 32.77 | 7.17 | 2.72 |
| C-L-13 | 0.08 | 33.74 | 6.41 | 2.43 |
| C-L-14 | 0.10 | 34.54 | 6.48 | 2.45 |
| C-L-15 | 0.10 | 33.27 | 6.13 | 2.32 |
| C-L-16 | 0.10 | 33.67 | 6.71 | 2.54 |
| C-L-17 | 0.10 | 41.10 | 5.78 | 2.19 |
| C-L-18 | 0.10 | 32.21 | 6.36 | 2.41 |
| C-L-19 | 0.15 | 64.29 | 6.20 | 2.35 |
| C-L-20 | 0.15 | 54.08 | 7.05 | 2.67 |
| C-L-21 | 0.15 | 44.59 | 8.12 | 3.07 |
| C-L-22 | 0.15 | 51.17 | 7.88 | 2.98 |
| C-L-23 | 0.15 | 53.82 | 7.87 | 2.98 |
| C-L-24 | 0.20 | 77.55 | 8.46 | 3.21 |
| C-L-25 | 0.20 | 79.94 | 7.08 | 2.68 |
| C-L-26 | 0.20 | 81.97 | 7.94 | 3.01 |
| C-L-27 | 0.20 | 81.88 | 7.01 | 2.65 |
| C-L-28 | 0.20 | 84.12 | 8.72 | 3.30 |
| C-L-29 | 0.25 | 101.00 | 10.21 | 3.87 |
| C-L-30 | 0.25 | 101.12 | 9.60 | 3.64 |
| C-L-31 | 0.25 | 99.70 | 10.89 | 4.12 |
| C-L-32 | 0.25 | 103.10 | 9.10 | 3.45 |
| C-L-33 | 0.25 | 100.74 | 8.11 | 3.07 |
| C-L-34 | 0.30 | 118.42 | 11.23 | 4.25 |
| C-L-35 | 0.30 | 119.74 | 9.41 | 3.56 |
| C-L-36 | 0.30 | 122.38 | 9.15 | 3.47 |
| C-L-37 | 0.30 | 118.89 | 8.70 | 3.30 |
| C-L-38 | 0.30 | 120.20 | 9.49 | 3.59 |
| C-H-11 | 0.10 | 37.52 | 6.96 | 2.68 |
| C-H-12 | 0.10 | 37.67 | 7.21 | 2.77 |
| C-H-13 | 0.10 | 38.17 | 7.49 | 2.88 |
| C-H-14 | 0.10 | 39.59 | 6.59 | 2.54 |
| C-H-15 | 0.10 | 40.56 | 6.95 | 2.67 |
| C-H-16 | 0.12 | 52.01 | 9.69 | 3.73 |
| C-H-17 | 0.12 | 50.35 | 9.44 | 3.63 |
| C-H-18 | 0.12 | 52.76 | 7.20 | 2.77 |
| C-H-19 | 0.12 | 51.87 | 9.07 | 3.49 |
| C-H-20 | 0.12 | 51.89 | 9.08 | 3.49 |
| C-H-21 | 0.14 | 65.54 | 9.21 | 3.54 |
| C-H-22 | 0.14 | 61.58 | 8.41 | 3.23 |
| C-H-23 | 0.14 | 61.10 | 8.72 | 3.35 |
| C-H-24 | 0.14 | 62.52 | 9.12 | 3.51 |
| C-H-25 | 0.14 | 63.03 | 9.57 | 3.68 |
| C-H-26 | 0.17 | 76.13 | 9.56 | 3.68 |
| C-H-27 | 0.17 | 79.87 | 8.00 | 3.08 |
| C-H-28 | 0.17 | 77.87 | 8.03 | 3.09 |
| C-H-29 | 0.17 | 78.29 | 7.87 | 3.03 |
| C-H-30 | 0.17 | 75.06 | 10.31 | 3.97 |
| C-H-31 | 0.20 | 86.77 | 9.76 | 3.75 |
| C-H-32 | 0.20 | 90.20 | 10.38 | 3.99 |
| C-H-33 | 0.20 | 88.78 | 9.82 | 3.78 |
| C-H-34 | 0.20 | 89.77 | 9.55 | 3.67 |
| C-H-35 | 0.20 | 89.04 | 11.48 | 4.41 |
| C-H-36 | 0.25 | 112.20 | 9.91 | 3.81 |
| C-H-37 | 0.25 | 116.03 | 9.26 | 3.56 |
| C-H-38 | 0.25 | 110.54 | 9.57 | 3.68 |
| C-H-39 | 0.25 | 110.99 | 9.88 | 3.80 |
| C-H-40 | 0.25 | 109.77 | 7.76 | 2.98 |
| C-H-41 | 0.30 | 122.36 | 10.35 | 3.98 |
| C-H-42 | 0.30 | 121.68 | 10.50 | 4.04 |
| C-H-43 | 0.30 | 124.14 | 10.47 | 4.03 |
| C-H-44 | 0.30 | 123.90 | 11.88 | 4.57 |
| C-H-45 | 0.30 | 126.95 | 11.37 | 4.37 |

**Supplementary** **Table S3**

Table S3. Results of dynamic splitting tests with BDs, where M represents mortar, and L and H refer to specimens with a thickness of 30 and 55 mm, respectively.

| **Specimen** | **Gas pressure (MPa)** | **Strain rate (s^−1^)** | **Tensile strength (MPa)** | **DIF** |
| --- | --- | --- | --- | --- |
| M-L-11 | 0.15 | 62.32 | 6.38 | 1.84 |
| M-L-12 | 0.15 | 57.51 | 6.84 | 1.97 |
| M-L-13 | 0.15 | 59.79 | 7.29 | 2.10 |
| M-L-14 | 0.15 | 59.29 | 8.46 | 2.44 |
| M-L-15 | 0.20 | 99.98 | 9.88 | 2.85 |
| M-L-16 | 0.20 | 96.02 | 7.67 | 2.21 |
| M-L-17 | 0.20 | 98.63 | 9.52 | 2.74 |
| M-L-18 | 0.20 | 98.67 | 8.97 | 2.59 |
| M-L-19 | 0.25 | 133.04 | 7.70 | 2.22 |
| M-L-20 | 0.25 | 131.41 | 8.77 | 2.53 |
| M-L-21 | 0.25 | 135.76 | 10.01 | 2.89 |
| M-L-22 | 0.30 | 152.86 | 9.63 | 2.77 |
| M-H-11 | 0.15 | 58.39 | 7.65 | 2.28 |
| M-H-12 | 0.16 | 54.99 | 8.30 | 2.48 |
| M-H-13 | 0.20 | 74.00 | 7.25 | 2.16 |
| M-H-14 | 0.20 | 94.88 | 9.24 | 2.76 |
| M-H-15 | 0.26 | 127.28 | 8.99 | 2.68 |
| M-H-16 | 0.25 | 124.82 | 9.73 | 2.90 |
| M-H-17 | 0.30 | 151.93 | 10.10 | 3.02 |

1. ^^ Corresponding author: Tel.: +86-29-82667864, Fax: +86-29-82669044

   E-mail address: fanxueling@mail.xjtu.edu.cn (X. Fan) [↑](#footnote-ref-2)
